# Study of the reasons for the consumption of each type of vegetable within a population of school-aged children

**DOI:** 10.1186/s12889-018-6067-4

**Published:** 2018-10-05

**Authors:** Laura Raggio, Adriana Gámbaro

**Affiliations:** 1 Food Department, Escuela de Nutrición (School of Nutrition), Universidad de la República, Montevideo, Uruguay; 2 Sensory Evaluation Area, Food Department, Facultad de Química (School of Chemistry), Universidad de la República, Montevideo, Uruguay

**Keywords:** Vegetables, Consumption, Children

## Abstract

**Background:**

Several studies have evaluated the existence of factors that influence the consumption of vegetables in children, such as family environment, daily exposure to one or several vegetables, parents’ consumption and consumption pattern and the way in which vegetables are prepared in the household, among others. The objective of this study was to investigate the reasons for consumption associated with each vegetable in school-aged children through a survey designed to be answered by the parents.

**Methods:**

A preliminary study with 162 parents was carried out on the consumption of vegetables in children aged 6 to 12 years. Based on the information obtained, a survey was designed with 14 phrases to investigate the reasons for the low consumption of each type of vegetable among school-aged children, which was answered online by 419 parents.

**Results:**

The results obtained allowed us to categorise the vegetables into 6 groups. ***Group A*** consisted of tomatoes, corn, pumpkin and carrots as the vegetables that children like to eat most. ***Group B*** contains the vegetables that are consumed mostly camouflaged in other preparations, such as onions and red peppers. ***Group C*** contains only cauliflower, which was negatively associated with senses, such as colour, smell and taste. This vegetable was never offered to children by a high percentage of parents. ***Group D*** consists of green vegetables: zucchini, spinach, chard and peas. Vegetables of this group are added to other foods and the child usually ingests them camouflaged or obliged. ***Group E*** consists of beetroot, lettuce and broccoli. Beetroot and lettuce were the vegetables parents reported were most often rejected by their children. This rejection, they stated, was due to sensory aspects, such as colour, texture and taste.

**Conclusions:**

The reasons for consumption among school-aged children depend on each type of vegetable and cannot be generalized. The sensory characteristics of the vegetable (mainly colour and flavour) and the habits of consumption in the family environment play a major role in children’s acceptance or rejection of vegetables.

## Background

Globalization and new lifestyles have led to major changes in eating patterns, which together with reduced physical activity have led to a significant increase in noncommunicable diseases (NCDs) [1]. NCDs are also known as chronic diseases because they are long lasting and usually evolve slowly. The main NCDs are obesity, cardiovascular diseases, cancer, chronic respiratory diseases and diabetes [2].

One of the main risk factors for NCDs, which is modifiable, is diet [3]. As part of a healthy diet, which should be low in fat, sugars and sodium, WHO suggests consuming at least 400 g of F&V per day [4]. In addition, fruits and vegetables are a rich source of vitamins and minerals, dietary fibre and other beneficial substances, such as phytosterols, flavonoids and other antioxidants [5]. Numerous investigations have shown the positive effects on the reduction of risk factors for NCDs associated with daily vegetable consumption in the recommended amounts [6, 7]. According to WHO data, an estimated 6.7 million deaths were due to inadequate intake of F&V in 2013 [8]. Worldwide, the 5 portions of fruits and vegetables recommended by WHO are not achieved [9–11]. The consumption in children and adolescents is also worrisome [12]. Children and adolescents in the United States consume 1 serving of fruit and 1.3 servings of vegetables per day [13]. In Germany, the average consumption of these foods in children between the ages of 3 and 17 years is below the recommended quantity. Only 12.2% of girls and 9.4% of boys consume the recommended 5 servings of fruits and vegetables per day [14]. In the UK, only 9% of children between 11 and 18 years of age are consuming the recommended quantities of fruits and vegetables every day, with vegetable consumption being especially low, with an average of one serving per day [15]. In Uruguay, only 24.4% of children and adolescents consume fruits and vegetables 5 or more times a day [16]. This low consumption of fruits and vegetables is consistent with that observed in the general population of the country in other surveys.

During the first years of a person’s life, the consumption of vegetables is very important. Their consumption in adequate amounts has been related to a healthier weight in childhood [17, 18] and in adulthood [19]. Secondly, healthy eating habits acquired during childhood tend to persist into adulthood [20–22].

Previous studies have evaluated the existence of factors that influence the consumption of food and in particular the consumption of vegetables. Some of the factors are knowledge, beliefs, cost, convenience and the sensory characteristics of vegetables [23–30].

In children, besides the factors mentioned, it should be added that their consumption is directly related to the family environment, daily exposure to one or several vegetables, parents’ consumption and consumption pattern and the way in which vegetables are prepared in the household, among others [31–35]. And in turn, the experience of trying new foods generates fear due to association with a negative sensory experience, and this could be especially important in the case of vegetables, since many of them have bitter tastes [36].

All the research works carried out to date study the factors linked to the consumption of vegetables by children in a generic way, considering vegetables as a homogeneous group of foods. The innovation in the present work is that each vegetable is studied individually, since the factors associated with consumption can differ between one vegetable and another.

The objective of this study was to go deeper into the reasons for consumption associated with each vegetable in school-aged children, through a survey designed to be answered by the parents.

## Methods

### Preliminary study

406 online invitations were sent to parents of children aged 6 to 12 years old. A total of 185 parents opened the survey and 162 complete it.The parents were recruited in eight educational centres in the city of Montevideo and its surroundings by means of an invitation sent to them from the school itself. The study was approved by the Human Beings Ethics Committee of the Facultad de Química, Universidad de la República. Written informed consent was obtained from each parent prior to data collection, and each parent was offered a copy of the consent form. The parents who agreed to participate provided a contact email to which they were sent a link to the survey.

The online questionnaire was developed using SurveyMonkey.com. The use of online questionnaires has been tested and found to be useful because of the ability to collect information from geographically distributed respondents, and because of the low cost compared with personal surveys. Another important feature is the convenience of the tool that allows access to the survey at any time [37].

A questionnaire with a list of the 18 most-consumed vegetables in the national market (tomatoes, lettuce, carrots, beetroot, eggplant, zucchini, onions, cucumber, pumpkin, spinach, chard, red peppers, cabbage, broccoli, cauliflower, green beans, peas and corn) was sent to the parents who agreed to participate in the survey [38]. They were asked to indicate their own consumption and their child’s consumption of each vegetable on the list by means of a structured scale of 7 points (1 = never, 2 = less than once a month, 3 = once or twice a month, 4 = several times a month, 5 = once or twice a week, 6 = several times a week, 7 = every day). For those vegetables which they replied their children ‘never consumed’, they were asked, through an open-ended question, to explain the reasons they believed their child did not consume them. At the end, the socioeconomic data of each parent (age, sex, marital status, education level, number of persons in the household, number of children in the household and age of the child) were collected.

### Design of the survey on reasons for vegetable consumption

Based on the information obtained in the preliminary study, a survey was designed to investigate the reasons for the low consumption of each type of vegetable among school-aged children (6 to 12 years old), consisting of 14 phrases (Table 1). For the construction of the phrases, no complicated terms or rare words were used. Short words were also used, making the questionnaire accessible.

**Table 1:** Phrases used in the survey to explore the reasons for the consumption of each type of vegetable

Phrase 1	My child usually eats:
Phrase 2	My child only eats camouflaged/disguised in other preparations:
Phrase 3	My child just eats when forced:
Phrase 4	My child used to eat it, but does not eat it now:
Phrase 5	I offered it to my child, but he/she never wanted to try it:
Phrase 6	My child does not eat it because I never offered it to him/her:
Phrase 7	My child does not eat it because he/she does not like its colour/appearance:
Phrase 8	My child does not eat it because he/she does not like its texture:
Phrase 9	My child does not eat it because he/she does not like its smell:
Phrase 10	My child does not eat it because he/she does not like its taste:
Phrase 11	I don’t know why my child does not like it:
Phrase 12	At home, we do not eat it because someone in the family does not like it:
Phrase 13	At home, we do not eat it because someone in the family has a health problem:
Phrase 14	At home, we do not eat it because I don’t know how to prepare/cook it:

To study whether the information obtained through the parents was representative of the children, individual face-to-face interviews were previously conducted with 15 families where the survey was applied. The children of the families interviewed were between 7 and 12 years old. In each family, the child and the parent were surveyed independently. The survey was conducted in their own home, which allowed the creation of an atmosphere of trust. It was requested that the parent who answered the survey was the one who was most present in the child’s meal instances (lunch/dinner) and/or the one who prepared the meals for the child. Of the 14 phrases, phrases 6, 11 and 14, were eliminated in the interviews made to children because it was not appropriate to ask them such questions. In the same way, the questionnaire was tested until an adapted final version was approved by the researchers.

### Survey on the reasons for the consumption of vegetables

The survey was sent online to the 185 parents to whom the exploratory survey had been sent and also distributed through social networks. The list of contacts included the parents who were invited to the preliminary study and also the staff (officials and teachers) of different faculties of the Universidad de la República. In total, 602 people opened the survey sent. At the beginning of the survey, it was stated that only parents of children between the ages of 6 and 12 years old should answer it and, if they had more than one child, they should answer it for only one of their children. The online questionnaire was developed using SurveyMonkey.com and consisted of the 14 phrases shown in Table 1. For each phrase, the parents received the list of the 18 vegetables used in the preliminary study, with the following cue: “CHECK ALL THE VEGETABLES YOU CONSIDER THIS PHRASE APPLIES TO”. At the end, the socioeconomic data of each parent (age, sex, marital status, education level, number of persons in the household, number of children in the household and age of the child) were collected.

### Statistical analysis

#### Preliminary study

An analysis of variance (ANOVA) was conducted on the parent’s consumption and child’s consumption data regarding the vegetables, the parent and child and their interaction as variation sources. The Tukey test was used to determine statistically significant (*p* ≤ 0.05) differences.

The answers obtained in the preliminary study of the open-ended question about the reasons the child ‘never’ consumed a certain type of vegetable were analyses qualitatively. According to Bengtsson, 2016 [39] and Erlingsso & Brysiewicz, 2017 [40], the analysis procedure of the raw data from the open-ended question of the surveys were transcribed to form categories or themes is a process of further abstraction of data at each step of the analysis; from the manifest and literal content to latent meanings. Analyses were performed individually by each of the members of the research team and the results generated were discussed further in detail by the research team before the final phrases were finally agreed upon by consensus.

#### Survey

The Chi square test was performed to determine significant differences (*p* ≤ 0.05) in the frequency distribution of socio-demographic variables between the participants of the preliminary study and the survey. The frequency of mention of each vegetable was determined for each of the 14 phrases, counting the number of times each vegetable was selected for each phrase. Cochran’s Q test was carried out to identify significant differences among vegetables for each of the phrases [41]. A correspondence analysis (CA) was performed on the frequency table considering chi-square distances. CA can be defined as a variant of principal components analysis, better suited for categorical data and especially contingency and frequency tables [42]. A hierarchical cluster analysis was performed on the answers obtained for each phrase to group the vegetables with similar answers. The formation of clusters was based on Ward’s aggregation criterion and Euclidean distances [42].

Statistical analyses were performed using XL-Stat 2017 software (Addinsoft, NY).

## Results

### Preliminary study

One hundred sixty-two parent complete the survey of the preliminary study. Table 2 shows the socioeconomic data of the parents who participated in the preliminary study. Most of the participants are between 30 and 45 years old and have a partner, which corresponds to the profile of parents with school-aged children.

**Table 2:** Socio-demographic data among respondents

		Preliminary study *n* = 162	Survey *n* = 419	*p*-value According to Chi.square test
Parent’s age	Mean	41 ± 6	42 ± 6	0.184
	18–30 years	6%	3%	
	30–45 years	88%	89%	
	over 51 years	6%	8%	
Gender	Male	11%	17%	0.072
	Female	89%	83%	
Marital status	Lives in partnership	80%	83%	0.397
	Lives alone	20%	17%	
Level of education	University professionals	57%	58%	0.827
	Tertiary education unfinished	43%	42%	
Persons in the household	2	5%	9%	0.243
	3–4	69%	68%	
	5 or more	26%	23%	
Children in the household	1	47%	45%	0.664
	2 or more	53%	55%	
Child’s age	Mean	9 ± 2	9 ± 2	

Table 3 shows the consumption of each type of vegetable of the parents and their children. A significant difference (*p* ≤ 0.05) was found in the frequency of consumption of the different vegetables, both in parents and in children. Parent consumption was significantly higher in 17 of the 18 vegetables studied. Corn was the only vegetable where the consumption of parents and children was similar. However, there is a great coincidence between the most and least consumed vegetables by both groups. The age and gender of the children did not significantly influence the consumption of vegetables, for which these results are not presented.

**Table 3:** Average values of the frequency of consumption of each type of vegetable for parent and children

Vegetable	Average parent consumption (7-point scale)	Average child consumption (7-point scale)	*p*-value
Cauliflower	1.6 ^a A^	1.4 ^a,b B^	0.0106
Cucumber	2.2 ^b A^	1.8 ^b,c B^	0.0011
Eggplant	2.5 ^b,c A^	1.7 ^a,b,c B^	< 0.0001
Green beans	2.5 ^b,c A^	2.0 ^c B^	< 0.0001
Broccoli	2.6 ^b,c,d A^	2.2 ^c B^	0.0023
Beetroot	2.7 ^b,c,d A^	2.0 ^c B^	< 0.0001
Cabbage	2.7 ^b,c,d A^	2.0 ^c B^	< 0.0001
Chard	3.1 ^d,e A^	2.7 ^d B^	0.0178
Peas	3.5 ^e,f A^	3.1 ^d,e B^	0.0035
Spinach	3.6 ^f,g A^	3.1 ^d,e B^	0.0004
Zucchini	3.8 ^f,g,h A^	3.2 ^d,e,f B^	< 0.0001
Corn	3.9 ^f,g,h,i A^	3.8 ^g,h A^	0.2582
Carrot	4.0 ^g,h,i A^	3.5 ^e,f,g B^	0.0001
Pumpkin	4.2 ^h,i,j A^	3.8 ^g,h,i B^	0.0143
Pepper	4.3 ^i,j,k A^	3.6 ^f,g,h B^	< 0.0001
Lettuce	4.6 ^j,k A^	3.0 ^d B^	< 0.0001
Onion	4.7 ^k,l A^	4.1 ^h,i B^	< 0.0001
Tomatoes	5.1 ^l A^	4.3 ^i B^	< 0.0001
*p*-value	< 0.0001	< 0.0001	

*For each column (parent or child consumption), the average followed by the same lowercase letter did not differed by Tukey test at 5% of probability*

*For each vegetable, the average followed by the same capital letter in the same line did not differed by Tukey test at 5% of probability*

Table 4 shows examples of the answers obtained in the open-ended question about the reasons the child did not consume that particular vegetable. For the vegetable *pumpkin*, there were no answers to the question about the reasons for non-consumption.

**Table 4:** Examples of answers to the open-ended question: Why does your child ‘never’ eat this vegetable?

Tomato	“because he does not want to try it”
Lettuce	“he says he cannot swallow it”, “it has no taste”, “because it is green”, “he has not developed a taste for it”, “because of its colour and texture”
Carrot	“It is not something we usually consume”, “I don’t often offer it to him because I don’t like it”, “only camouflaged with pumpkin”
Beetroot	“because of its taste”, “because of its strong taste”, “the colour makes him reject it”, “the family does not eat it”
Eggplant	“we do not usually eat it”, “I know only a few preparations that include it”, “because it is bitter”, “only in ‘milanesas’”, “its taste is a little spicy”
Zucchini	“he does not accept green vegetables”, “he cannot find its taste”, “camouflaged, sometimes”, “we do not eat it”
Onion	“because of its strong smell”, “because of its taste”, “only in recipes”, “we do not eat it”, “if he sees it, he does not eat it”
Cucumber	“because of its appearance”, “it is not included in family meals”, “we do not eat it”, “because of lack of habit”
Spinach	“he does not like green ones”, “we do not usually eat it at home”
Chard	“I do not buy it because it produces gas and it’s bitter”, “we do not usually eat it”, “we do not like its taste”, “just camouflaged as an ingredient in recipes”
Pepper	“strong taste”, “bitterness”, “only in recipes”, “he eats it by obligation”
Cabbage	“we do not eat it”, “very different taste”, “someone in the family does not like it”, “because it’s green”
Broccoli	“it’s not included in the diet”, “because of its taste”, “my daughter used to eat it frequently until she got tired of it”, “he does not like it”
Green beans	“because of its taste”, “we do not eat it”, “because of its appearance”, “he has not adapted to it yet”, “it is green”
Peas	“I do not buy canned food”
Corn	“because it is sweet”, “only in recipes”
Cauliflower	“we do not eat it”, “he dislikes its smell and taste”, “I do not know how to prepare it”, “he does not like its taste or its smell”

### Survey

In the face-to-face interviews with the 15 families, in order to test the comprehension of the phrases and verify that the parents’ responses were representative of the children, more than 95% agreement was obtained between parents’ and children’s responses.

The survey was opened by 602 parents, and fully answered by 419 parents (69.6%). Table 1 shows the socioeconomic data of the parents who participated in the survey. These participants were also mostly women, between 30 and 45 years old, in a relationship and with university studies finished. According to the Chi square test, no significant difference (*p* > 0.05) was found between the socioeconomic data of the parents who participated in the preliminary study and those who did it in the survey.

All the phrases except number 13 received responses greater than 10% on at least one of the vegetables. Phrase 13 (“at home we do not eat it because someone in the family has a health problem”), was eliminated from the analysis due to its low number of answers (less than 2%).

The results obtained in the survey are described below. The data in parentheses indicate the percent of respondents that marked that vegetable for a particular phrase. For the group of parents surveyed, their children usually consume tomatoes (70%), carrots (53%), pumpkins (59%) and corn (67%) because they like it. Red peppers (37%), onions (41%), zucchini (25%), carrots (24%), spinach (22%) and chard (16%) are consumed if the children are not aware that they are eating those vegetables. Zucchini and carrots are included in this category, but with a lower contribution (25%).

Zucchini (10%) was considered as the only vegetable that the children ate because their parents forced them. There was a very low response rate to this phrase. One might conclude that “obliging” a child to eat a vegetable is frowned upon in today’s society, so parents may not have used this phrase much to explain the consumption of some vegetables.

As for those vegetables that their children consumed in the past, but no longer consume, we can find green vegetables, such as zucchini (13%), spinach (12%) and broccoli (10%), as well as pumpkin (14%). The vegetables that children were offered, but never wanted to try were beetroot (25%), eggplant (24%), cucumber (22%) and a broad list of green vegetables, such as lettuce (20%), green beans (20%), broccoli (19%), cabbage (17%) and peas (13%).

Among the vegetables that parents never offered their children, we find cauliflower (43%), cabbage (23%), cucumber (21%), eggplant (18%), green beans (17%) and broccoli (16%). This is related to those that one of the parents does not consume or does not know how to prepare (phrases 12 and 14).

The vegetables that are not consumed because of their colour are eggplant (22%), cauliflower (19%), beetroot (17%) and broccoli (17%), followed by green ones, such as green beans (14%), lettuce, spinach and chard (13%), cabbage (12%), zucchini and cucumber (11%). Of those that are not consumed because of texture, only lettuce appears in 12% of answers. Those that are disliked because of their smell are cauliflower (22%) and broccoli (17%). Those disliked because of their taste are eggplant (19%), beetroot (17%), cauliflower (16%), broccoli, cabbage (15%), pepper and cucumber (14%).

The parents surveyed provided a low response rate for phrase 14. It was only stated that they do not know how to cook cauliflower (12%), which agrees with the fact that it is one of the vegetables that was never offered by the parents (43%) and someone in the family does not like it (40%).

For a better visualization of the relationship between vegetables and each phrase, a correspondence analysis was performed. Correspondence analysis decomposes the overall inertia (the correlation between the data points of the variables in the analysis) by identifying a small number of dimensions that represent all the locations of the data point’s well [41]. In practice a two-dimensional solution (dimension 1 and dimension 2) will represent the data well.

**Fig. 1:**
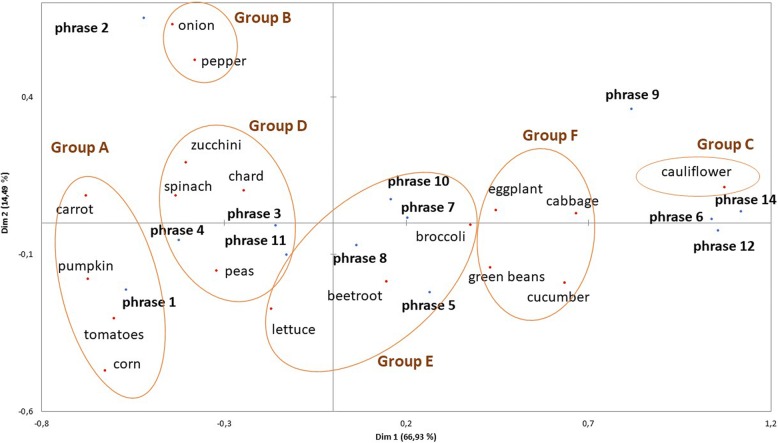
Results of the correspondence analysis (CA). The first two dimensions (dim1 and dim2) of the CA accounted for 81.4% of the variance of the experimental data

The results are presented in Fig. 1. Dimension 1 and dimension 2 explained 81.42% of the variance. Subsequently, a hierarchical cluster analysis was applied to the answers obtained for each phrase, which allowed the categorising of the vegetables into 6 groups.

***Group A*** consisted of **tomatoes**, **corn**, **pumpkin** and **carrots**. These are the vegetables that children like to eat most. In spite of this, pumpkin is also one of the most mentioned vegetables as the one they used to eat, but do not eat now. This shows signs of the evolution in children’s consumption habits during growth. There is no mention of rejection due to colour, which suggests that children like the colour orange. In addition, carrot is another vegetable that children like to eat although it is also one of those added in recipes and ingested ‘camouflaged’.

***Group B*** contains the vegetables that are consumed mostly ‘camouflaged’ in other recipes, such as **onions** and **red peppers**. Parents relate their rejection with sensory characteristics, such as taste and colour.

***Group C*** includes only **cauliflower**, which was negatively associated with the senses because of its colour, smell and taste. This vegetable was never offered to children by a high percentage of parents while in other cases, it was offered, but children never wanted to try it. Both reasons are strongly associated with the high percentage of parents who said that one family member did not like it. In addition, they reported not knowing how to cook it.

***Group D*** consists of green vegetables: **zucchini**, **spinach**, **chard** and **peas**. Of this group, zucchini, spinach and chard are added in recipes and the child usually ingests them ‘camouflaged’. In addition, zucchini was the only one that was declared to be consumed out of force. In turn, zucchini and spinach were considered to be vegetables that children used to consume before, but no longer consume. They relate this rejection with sensory aspects, mainly colour and taste. Chard is rejected because of its colour, and peas are rejected because of their taste.

***Group E*** consists of **beetroot**, **lettuce** and **broccoli**. Beetroot and lettuce are the main vegetables reported by parents which were most offered to their children that they did not want to try. This rejection, they stated, is due to sensory aspects such as colour and taste for beetroot. In the case of lettuce, the parents believed that their children did not want to try it mainly for sensory reasons, such as colour, texture and taste. Broccoli is a vegetable that according to parents is not consumed for several reasons: children used to consume it and now they do not, it was offered to them, but they never wanted to try it, it was offered to them and it is rejected because of sensory factors like colour, smell and taste.

***Group F*** consists of **eggplant**, **cucumber**, **cabbage** and **green beans**. This group of vegetables stands out because these vegetables have never been offered to children by a high percentage of parents, mainly because someone in the family did not like them. Eggplant was reported by parents as the vegetable they most offered their children that they did not want to try. Also, children did not eat it because it was not offered to them. Reasons for rejection are related to sensory aspects, such as taste and colour. As for the cucumber, its low consumption is explained by its colour and taste, but also because someone in the family did not like it. Many parents declare that they offered their son/daughter cabbage, but he/she did not want to try it. There are sensory aspects that generate such rejection such as the colour and taste. Green beans were offered, but the children never wanted to try them, just like the vegetables of this group. This is due to sensory aspects that generate rejection, mainly colour and taste.

## Discussion

### Preliminary study

According to the socioeconomic data of the parents who participated in the preliminary survey, high percentage of women answered the survey. In the Uruguayan culture, it is usually the mother who prepares the meals for the child. Similar results were reported [43] out of a total of 582 parents surveyed on their mealtime actions, their children’s food intake and the characteristics of the family meals. Eighty five percent of respondents were the mothers. For the UK households, a comparative participation of women was reported [44].

### Survey

A similar correlation between parents’ and children’s responses was found in [45], as we found in the face-to-face interviews with the 15 families.

A total of 69,6% of the parent that opened the survey, answer it. So we have a much higher response compared with reported by other authors [37–46].

Regarding the data on the consumption of vegetables obtained in the preliminary study, the coincidence between the groups of vegetables most consumed by the parents and by the children stands out. Similar results were reported [27], in which the consumption between parent and child correlated with a higher consumption rate in those children whose parents consume more of this type of food. As has been emphasized by numerous publications, parents play an important role in child and adolescent eating behaviour, and also peer influence is highlighted [47, 48]. In the same way, it has been found that the food preferences and eating patterns that develop in early childhood and adolescence do not increase later in adulthood, so it is very important to have healthy preferences and a high consumption of healthy foods incorporated from early ages [49–51].

The results obtained from the survey designed on the reasons for this low consumption of vegetables showed a low response for the phrase related to “forcing them to eat certain vegetables” and also for my child only consumes ‘camouflaged’ in other recipes, resulting in both situations being seen as something socially negative. Forcing children to eat has been associated with aversion [52] and a reduced intake of these foods [53, 54]. Furthermore, the context of consumption of certain foods influences their total intake [55]. In particular for vegetables, the context should be as enjoyable as possible and forcing a child to consume a certain food will not encourage consumption. According to results obtained [27], children of parents who tend to put stronger pressure on their children to eat vegetables or who act as negative role models more often ate fewer vegetables. In view of the results obtained, it is encouraging to know that a low amount of vegetables was associated with the phrases ‘my child only eats when forced’ and ‘my child only eats when disguised’ preventing the future development of a negative relationship with the consumption of such vegetables.

Food rejection in children is presented in two forms: Food neophobia and picky/fussy eating behaviour. These two forms of food rejection are age-related and temporary behaviours, so they are important to consider since our surveyed children are between 6 and 12 years old [56, 57]. According to some authors, ‘pickiness’ normally reaches its peak when the child is between 3.5 and 5.5 years old and then it decreases gradually [58]. However, the range of pickiness among children is large, and almost 20% of children between 8 and 12 years of age can still be considered picky eaters, meaning that the variety of their diets may be considered insufficient [56, 59]. Pickiness has disadvantages because it is related to insufficient vegetable and fruit intake, and a healthy diet requires varied food intake [60]. This vegetable rejection behaviour explains the answers obtained from the phrase ‘my child used to eat it, but he/she does not do it now’ (phrase 4), especially for the group of green vegetables.

The vegetables mentioned for the phrases “I offered it to him/her, but he/she never wanted to try it” (phrase 5) and “my son used to eat it, but he does not do it now” (phrase 4) has reasons strongly associated with sensory aspects, colour in particular. The palatability and taste of the food are said to be shaped by the colour of the food [61]. From the frequency of vegetable consumption of parents and children and the response to the phrase ‘my child usually eats it’, we discover that the most consumed vegetables by children are those which are red and orange, and also have a sweeter taste, such as tomatoes, carrots, pumpkins and corn.

The importance of the relationship between parents and children in the consumption by children of certain types of vegetables (such as cucumber, broccoli, cauliflower and green beans) is evidenced through the answers obtained from the phrases ‘my child does not eat it because I never offered it to him/her’, ‘at home we do not eat it because I do not know how to prepare it/cook it’ and ‘at home we do not eat it because someone in the family doesn’t like it’. According to a model presented, the factors that affected in a postitive way the intake of vegetables were availability and accessibility [62]. When parents have more fruits and vegetables available in the home, child consumption improves [63]. The reason they never offered their children certain vegetables may be because they were unaware of the nutritional benefits of their consumption [64]. If parents as well as caregivers had such information, they could teach them and create an opportunity for children to be informed consumers so that they could make decisions about their own nutrition [65]. The concept of responsible parenting, particularly in relation to food, is reflected in the relationship between the caregiver and the child. This is one way healthy habits can be promoted [66, 67].

The most obvious reason children do not want to eat a certain food is because they do not like the taste of it [68]. However, children do not only reject food because of the flavour, they can also dislike the texture, colour/appearance and smell of the food. That is why these sensory aspects were evaluated in independent phrases (phrases 7, 8, 9 and 10) in order to accurately assess the sensory reasons children rejected certain vegetables. The results obtained aligned with those obtained by other researchers for the vegetables in general. There is a widespread rejection of the green colour and bitter tastes found in most vegetables [61]. In addition, it was reported that the low preference has been attributed to our innate aversion to bitter tastes [69]. The texture of vegetables was also mentioned as a major reason for the acceptance or rejection in children [30, 70], and the modifications suffered by the texture of vegetables according to their cooking methods [31]. These researchers reported that the sensory reasons children do not prefer vegetables are flavour (sour/acid), texture and appearance.

Knowing certain preparation techniques and how to cook the vegetables has been studied by several authors [31, 71–73]. Some of the most used techniques with vegetables are blending, mixing, mashing or seasoning, and it has been seen that knowing how to apply them properly by the parents increases the consumption of vegetables. Parents surveyed reported a low response level for phrase 14 (“I do not know how to prepare them”). Only 12% stated that they did not know how to cook cauliflower, which is consistent with the fact that it is one of the vegetables that was never offered by the parents (43%) and someone in the family did not like it (40%).

Finally, some limitations of this study should be considered. First, data collection was done through online surveys, so socially correct responses could have influenced the results. Secondly, we used a convenience sample comprising parents who agreed to participate, therefore selection bias could be a problem. Only the 18 most consumed vegetables in the local market were considered for the study. Other vegetables (such as arugula, radish, celery, artichoke, turnip, watercress, avocado, etc.) were not included in this study.

## Conclusions

The present study shows that the reasons for consumption among school-aged children depend on each type of vegetable and cannot be generalized. The sensory characteristics of the vegetable (mainly colour and taste) and the habits of consumption in the family environment play a major role in school-aged children’s acceptance or rejection of vegetables. Actions to increase vegetable consumption among children should aim to encourage parents to act as role models and to raise awareness of strategies to change their child’s eating behaviour. Future studies should aim to determine the reasons for consumption of each type of vegetable in other populations in order to investigate the results obtained and also to study if changes in the determinants of vegetable intake in the parents could affect the behavior of the child.
